# The RGS gene *loco *is essential for male reproductive system differentiation in *Drosophila melanogaster*

**DOI:** 10.1186/1471-213X-8-37

**Published:** 2008-04-03

**Authors:** Leeanne McGurk, Stephen Pathirana, Kathleen Rothwell, Thorsten Trimbuch, Paolo Colombini, Fengwei Yu, William Chia, Mary Bownes

**Affiliations:** 1 Medical Research Council Human Genetics Unit, Western General Hospital, Edinburgh, EH4 2XU, UK; 2 European Commission Enterprise and Industry Director General, BREY 07/318 45, Brussels 1040, Belgium; 3 University of Edinburgh, Institute of Cell Biology, Edinburgh EH9 3JR, UK; 4 Charite – Universitaetsmedizin Berlin, Centrum fur Anatomie, Insitut fur Zell- und Neurobiologie, Charitéplatz 1, 10117 Berlin, Germany; 5 Via Giovanni Guareschi, 16, 41010 Cognento, Modena, Italy; 6 Temasek Life Sciences Laboratory and Department of Biological Sciences, National University of Singapore, 117604, Singapore

## Abstract

**Background:**

The *loco *gene encodes several different isoforms of a regulator of G-protein signalling. These different isoforms of LOCO are part of a pathway enabling cells to respond to external signals. LOCO is known to be required at various developmental stages including neuroblast division, glial cell formation and oogenesis. Less is known about LOCO and its involvement in male development therefore to gain further insight into the role of LOCO in development we carried out a genetic screen and analysed males with reduced fertility.

**Results:**

We identified a number of lethal *loco *mutants and four semi-lethal lines, which generate males with reduced fertility. We have identified a fifth *loco *transcript and show that it is differentially expressed in developing pupae. We have characterised the expression pattern of all *loco *transcripts during pupal development in the adult testes, both in wild type and *loco *mutant strains. In addition we also show that there are various G-protein α subunits expressed in the testis all of which may be potential binding partners of LOCO.

**Conclusion:**

We propose that the male sterility in the new *loco *mutants result from a failure of accurate morphogenesis of the adult reproductive system during metamorphosis, we propose that this is due to a loss of expression of *loco c3*. Thus, we conclude that specific isoforms of *loco *are required for the differentiation of the male gonad and genital disc.

## Background

Many hormones and neurotransmitters act by binding to G-protein-coupled receptors (GPCRs) which transduce the signal via second messengers such as cAMP. The heterotrimeric G proteins comprise of one member from each of the Gα, Gβ and Gγ families. In the absence of an external signal the GPCRs are associated with an inactive heterotrimer complex, Gα-GDP/Gβ/Gγ. When a specific ligand binds a GPCR, the intrinsic nucleotide exchange factor (GEF) activity is activated; the resultant Gα-GTP subunit dissociates from Gβ/Gγ, leaving the Gβ/Gγ heterodimer and the Gα-GTP to spread the signal to downstream target molecules. The GTP is slowly hydrolysed by Gα, and the Gα-GDP then returns and binds to the Gβ/Gγ complex rendering the receptor inactive. RGS proteins (Regulator of G-protein signalling) are a family of GTPase activating proteins (GAP) that trigger the intrinsic GTPase activity of the Gα subunits [1,2]. Although a great deal is known about the regulation of G-protein-coupled receptor signalling in a variety of organisms [3,4] less is known in *Drosophila *and more importantly the involvement of G-protein-coupled receptor signalling in developmental decisions.

**Figure 1:**
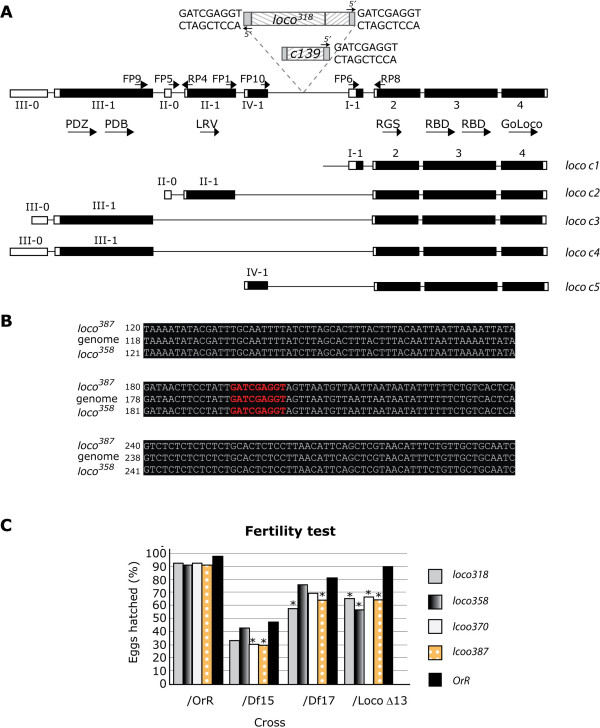
**The *loco *gene and its effect on male fertility**. **A**: The *loco *gene is comprised of nine exons. The final three exons, exon 2, 3 and 4 are common to all *loco *transcripts whereas the 5'exons are alternatively spliced to give rise to five different *loco *transcripts; *loco c1 *(AF130745), *loco c2 *(AF130744), *loco c3*, (AF245455), *loco c4 *(CG5248 PC) and *loco c5 (*AI944917). The exon numbers are shown below the genomic sequence and the conserved domains of the LOCO protein and the primers used to identify the transcripts in the testis and pupae are marked above the appropriate exons. Black regions indicate coding sequence, white regions represent untranslated regions. The P element in the original P insertion line, c139, is 322 bp upstream of exon 2. The *loco*^*318 *^mutant contains two P elements (or one P element and a partial P element) in reverse orientation 322 bp upstream of exon 2. The P elements of *loco*^*318 *^are flanked by a 9 bp duplication of genomic sequence. **B**: The sequence surrounding the P element insertion site in c139 was sequenced in *loco*^*358 *^and *loco*^*387 *^and aligned to the genomic DNA using ClustalW [36] and BoxShade [37]. No deletion was observed. The region of genomic DNA that is duplicated in *loco*^*318 *^is highlighted in red. **C**: Hemizygous flies, containing the *loco *mutation and the deficiency chromosome (*Df(3R)15CE1 (Df15), Df(3R)17D1 (Df17), or loco*^*Δ13 *^) or the wild-type, *OrR*, chromosome, were crossed with *OrR*, *Df15, Df17 or loco*^*Δ13 *^virgin females. The ratio between the total number of eggs laid and the number of eggs which hatched is represented as a percentage. Chi squared values were calculated by comparing the heterozygous male semi-steriles to *OrR *and the hemizygous mutants to the heterozygous deficiency lines. Chi squared values over 6.64 suggest that the reduction in fertility is due to the two lines being closely linked. Asterisks indicate a statistically significant reduction.

In *Drosophila *nine genes encoding for RGS proteins have been identified [5], however protein function has only been studied in three of them, *axin, gprk2 *and *loco*. *Daxin*, the *Drosophila *orthologue of *axin *[6], is a scaffold protein, that in the absence of Wnt signaling, negatively regulates cytosolic Armadillo by aiding its proteosome-dependent degradation [7-9]. The negative regulation of Armidillo by axin is inhibited by the interaction of Axin with the Gαs subunit of Prostaglandin E2-stimulated in colon cancer cells [10]. Gprk2, G-protein-coupled receptor kinase 2, maintains cAMP levels in the ovary and is required for embryonic anterior patterning [11,12]. The *Drosophila loco *gene encodes a number of LOCO protein isoforms, all of which contain the RGS domain and also a GoLoco motif that acts as a Guanine nucleotide dissociation inhibitor (GDI), (Figure 1A). Previous studies suggest that LOCO might play an important role during early *Drosophila *development. For example during asymmetric cell division of the *Drosophila *neuroblast, LOCO may act as a GTPase activating protein of the Gαi protein via its RGS domain as well as a (GDI) through its GoLoco motif [13]. In the *Drosophila *embryo *loco *is essential for the formation, extension, and migration of glial cells, and plays a role in asymmetric cell division of *Drosophila *neuroblasts [13,14]. Rare adult flies lacking the *loco c1 *and *loco c2 *transcripts have locomotive defects [14]. Previously, we showed that *loco *is expressed in the nurse cells and in specific subsets of the follicle cells of the *Drosophila *egg chamber, and that it is required for cytoplasmic dumping during oogenesis and for dorsal-ventral axis formation of the egg chamber and embryo [15].

The *Drosophila loco *gene encodes a number of isoforms of an RGS protein (Figure 1A). *loco c1 *and *loco c2 *were the first transcripts to be identified and were found to be differentially expressed during embryogenesis [14], subsequently we identified a third transcript, *loco c3*, and showed that it was required for egg and embryo development [15]. The sequence data on *loco c3 *has been extended to show that more sequence, including a start site, was upstream of the original start site identified for *loco c3*. The two start sites are in frame, but neither has been shown to be functionally active [13]. To remain in line with the published nomenclature, we will call this extended transcript *loco c4*.

Until now nothing was known about the involvement of RGS in G protein signalling in the male reproductive system, we therefore set out to determine if LOCO was also essential for male development. We isolated male fertility mutants from a P element mobilisation screen [15] and have shown that the male sterility is due to mutations mapping in the *loco *gene. Gene expression analysis has not only identified disrupted gene expression in the *loco *mutant lines but it has also revealed a fourth *loco *transcript required for correct male development. This alongside the phenotypic analysis of the semi-sterile males suggests a role for *loco *in the differentiation of the testis from the male gonads and genital discs. Furthermore we analyse *loco *expression in male gonads and the male adult reproductive tissue in both wild-type and *loco *mutant lines.

Finally, RGS proteins, such as LOCO, negatively regulate signalling mediated by G-protein coupled receptors, by reducing the time that the Gβ/Gγ subunit is available to signal. However with an additional GoLoco motif, LOCO can also increase the initiation rate of G protein signalling [13]. LOCO may well regulate this signalling pathway in the follicle cells of the *Drosophila *egg and glial cells of the embryo by binding to the *Drosophila *Gαi subunit [14,16,17]. In order to analyse the presence of Gα-proteins, which could potentially interact with LOCO in the *Drosophila *testis, we undertook a candidate PCR approach and identified a further two Gα subunits expressed in the testis.

## Results

### Screens for male sterility

Previously we carried out a P element mediated mutagenesis screen using a P element located between exons II-1 and I-1 of the *loco *gene (Figure 1A) [15]. We established that perfect excision of this element led to fully viable fertile lines, indicating that there were no other mutations in the stock. Most of the 399 lines that we generated were homozygous lethal and many of the viable lines produced very few homozygous adults, indicating a requirement for *loco *during development. Many of the lines, which generated some adults, also showed reduced fertility in females. Complementation analysis of the 399 lines showed that the mutants fell into two different complementation groups, however, two mutant lines fell into both complementation groups. The complex splicing of *loco *transcripts makes it likely that both of the complementation groups affect different essential transcripts of the *loco *gene.

In this study we wanted to determine if any of the semi-lethal lines which generated some adult progeny were male sterile. We found that 27 of the *loco *mutant lines generated a few adult males. The fertility of the rare adult males was investigated by crossing the homozygous *loco *mutant males to *OrR *virgin-females. Five lines produced none or very rare progeny; *loco*^*318*^, *loco*^*358*^, *loco*^*455 *^, *loco*^*370*^, and *loco*^*387*^. The first three of these are red-eyed lines, which have either resulted from a partial excision or from a mobilised P element. The latter two lines are white eyed and therefore lack at least part of the original P insertion and possibly some flanking genomic DNA. One of these lines (*loco*^*455*^) subsequently ceased producing any adult males and was not further investigated. Complementation analysis showed that the mutations of the semi-sterile males fell into the same complementation group (Table 1). The rare heteroallelic males produced in these experiments generated very few progeny when crossed to *OrR *females (this includes line *loco*^*455*^).

**Table 1:** Complementation tests for male fertility

**Male**	**Average number of progeny produced per cross**
*loco*^*358*^*/loco*^*318*^	14
*loco*^*387*^*/loco*^*318*^	3
*loco*^*387*^*/loco*^*358*^	3
*loco*^*370*^*/loco*^*358*^	8
*loco*^*370*^*/loco*^*318*^	49
*loco*^*T1*^*/loco*^*318*^	5
*loco*^*Δ113*^*/loco*^*387*^	7
*loco*^*Δ113*^*/loco*^*358*^	2
*loco*^*Δ113*^*/loco*^*318*^	68

Flies heterozygous for each of the male semi-sterile lines and one of the *Df *lines that affects loco (*loco*^*Δ13*^) were crossed and rare heteroallelic mutant males were collected. Groups of 2–3 males crossed with 3–4 *OrR *females would normally generate several hundred progeny if the males are also *OrR*. Each experiment was repeated twice. No adult males were obtained for *loco*^*455*^.

None of the *loco *mutants heterozygous with *OrR *showed significantly reduced male fertility, indicating that the semi-sterile *loco *mutant males were fully recessive (Table 2). To further characterise the *loco *mutants we analysed male fertility in two other strains lacking *loco*. The two deficiency strains used were *Df(3R)15CE1 *(*Df15*), which lacks the cytogenetic region 93F-;94C-94D, and *Df(3R)17D1 *(*Df17*), which lacks 93E-94C, both lack the *loco *gene which is at position 94B6-94B8. The deficiency lines were crossed to *OrR *and the resulting heterozygous males were crossed to *OrR *to assess the effect of the mutation on fertility (Table 2). Only *Df15/+ *showed a significant reduction in fertility when compared to *OrR*, this is likely to be due to the loss of genes present in the region 94C-D as *Df17/+*, when crossed to *OrR*, did not show any significant reduction in male fertility (Table 2). Furthermore *loco*^*Δ113*^, a mutant strain, which was previously reported to lack part of the *loco *gene and 7 kb of downstream sequence [14] also showed no significant reduction in fertility when crossed to *OrR *(Table 2). This suggests that heterozygous loss of *loco *does not affect male fertility.

**Table 2:** Complementation of male semi-sterile lines with deficiency lines

**Cross**	**Total Eggs**	**Total Larvae**	**% viability**	**Chi**^**2**^
*OrR*	217	214	98.6	/
*loco*^*318*^*/OrR*	299	273	92.3	0.32 NS
*loco*^*358*^*/OrR*	237	217	91.6	0.33 NS
*loco*^*370*^*/OrR*	240	223	92.9	0.22 NS
*loco*^*387*^*/OrR*	313	285	91.1	0.51 NS
*OrR/Df15*	435	208	47.8	45.25 **S**
*OrR/Df17*	407	332	81.6	3.46 NS
*OrR/loco*^*Δ13*^	275	249	90.5	0.49 NS
*loco*^*318*^*/Df15*	366	121	33.1	5.86 NS
*loco*^*318*^*/Df17*	791	459	58.0	17.67 **S**
*loco*^*318*^*/loco*^*Δ13*^	1062	693	65.3	24.70 **S**
*loco*^*358*^*/Df15*	259	112	43.2	0.31 NS
*loco*^*358*^*/Df17*	368	280	76.1	0.39 NS
*loco*^*358*^*/loco*^*Δ13*^	228	131	57.5	9.94 **S**
*loco*^*370*^*/Df15*	250	76	30.4	6.90 **S**
*loco*^*370*^*/Df17*	403	284	70.5	1.98 NS
*loco*^*370*^*/loco*^*Δ13*^	731	491	67.2	14.23 **S**
*loco*^*387*^*/Df15*	755	226	29.9	22.06 **S**
*loco*^*387*^*/Df17*	569	368	64.7	6.75 **S**
*loco*^*387*^*/loco*^*Δ13*^	873	570	65.3	35.65 **S**

Heterozygous flies, containing the *loco *mutation and the deficiency chromosome (*Df(3R)15CE1*, *Df(3R)17D1*, *loco*^*Δ13*^) or the *OrR*, chromosome, were crossed with *OrR*. The ratio of eggs laid and eggs hatched is represented as a percentage. Significance was calculated by calculating Chi^2 ^values between heterozygous mutant males and *OrR*, and the hemizygous mutants to the heterozygous deficiency lines. Chi^2 ^> 6.64 suggests the two lines are closely linked. S: significant, NS: not significant.

In order to assess whether our *loco *mutants were in the same or different complementation groups to the deficiency lines [14], the four *loco *mutant lines were crossed to each of the above mentioned deficiency lines. All heteroallelic mutants had reduced fertility when compared to the heterozygous mutants (Figure 1C). Despite all of the heteroallelic *loco *mutants showing reduced fertility not all were a statistically significant reduction (Table 2). However all of the mutant *loco *alleles (*loco*^*318*^, *loco*^*358*^, *loco*^*370*^, and *loco*^*387*^) hemizygous with the *loco*^*Δ113 *^allele, showed a statistically significant reduction in male fertility (Table 2 and Figure 1C). This not only suggests that the mutations isolated here fall into the same complementation group but, that the mutations we have isolated reside within the *loco *gene.

### Expression of *loco *in the testis

**Figure 2:**
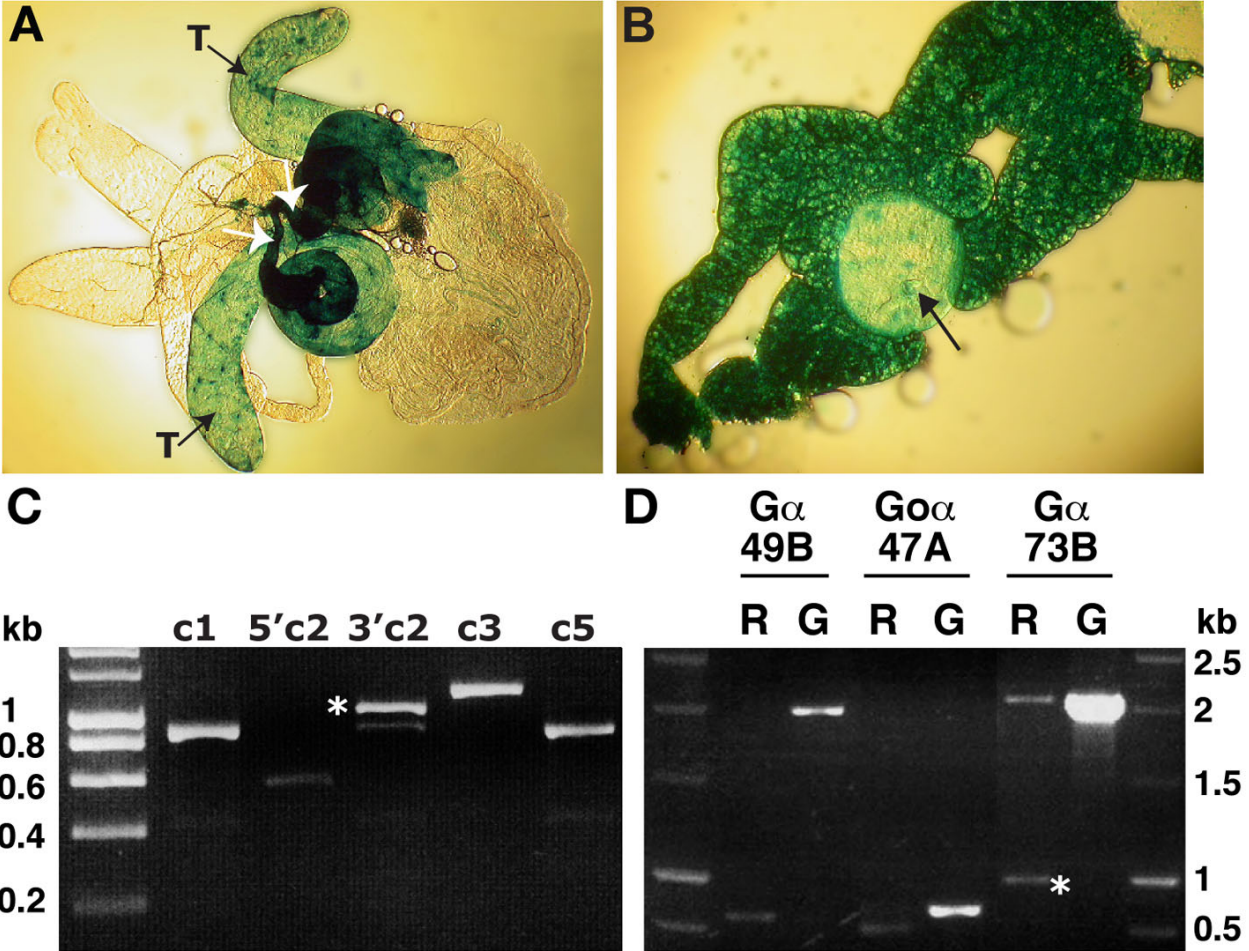
**Expression of *loco *in the male gonads**. **A**: A *UAS-lacZ *reporter line revealed that *loco *was strongly expressed in the adult testes (T) and seminal vesicles (white arrows). **B**: The β-galactosidase reporter revealed that *loco *was expressed in the male gonad (black arrow) and in the surrounding fat body tissue. **C**: The primers shown in Figure 1A were used to establish that *loco c1*, *loco c2*, *loco c3 *and *loco c5 *were expressed in *OrR *testes. The band marked with an asterisk is the true PCR product of the 3' end of *loco c2 *(3'c2) as determined by sequence analysis. **D**: Several G-protein transcripts were detected in *OrR *testis. The asterisk indicates the true PCR product of expressed Gα73B as determined by sequence analysis. R: RNA, G: genomic DNA.

The semi-sterile males obtained from the *loco *mutants isolated in this screen suggested that LOCO may have a role within the testes. To investigate whether *loco *was expressed in the male gonads a *UAS*-*lacZ *reporter strain was crossed to the original P insertion strain (c139), which contained a *GAL4 *insertion in the *loco *gene [15]. The resulting male adult gonads were stained for β-galactosidase activity. The testis and seminal vesicles showed very strong *lacZ *expression (Figure 2A). The *loco *gene is alternatively spliced to give rise to several isoforms *loco c1*, *loco c2*, *loco c3*, and *loco c4 *[13-15]. Upon database searching we revealed that there was a fifth transcript, isolated from an adult EST testis library (Accession Number AI944917). The sequence had no conserved domains relating to LOCO and mapped to the genomic region located in the intron between exon II-1 and exon I-1 (Figure 1A). This suggested that there was an additional transcript, not previously described, but expressed in the testis. This new exon has been labelled exon IV-1 and the transcript produced is *loco c5 *(Figure 1A).

In order to analyse the expression of the various *loco *splice variants in the *Drosophila *testis primers were designed to specifically amplify *loco c1*, *loco c2*, *loco c3 *and *loco c5*. It should be noted that the primers used to amplify *loco c3 *could not discriminate between *loco c3 *and *loco c4*, furthermore it is possible that these two transcripts form the same transcriptional unit. RT-PCR revealed that *loco c1, loco c2, loco c3, and loco c5 *were expressed in the *OrR *male testis (Figure 2C). Detection of *loco c2 *using the primer pair FP1 and RP8 (Figure 1A) produced a specific product at approximately 1 kb and two non-specific bands at lower molecular weights (Figure 2C, 3'c2). Cloning and sequencing revealed that the 1 kb band was specific to *loco c2*, the 0.9 kb band aligned to CaBP1 (CG5809) and the 0.4 kb band aligned to myosin binding subunit (CG32156). We also show later in the paper that the novel transcript identified from the testis EST library is not unique to the testis, as it is also expressed in pupae. Thus what we have identified and described is a further novel *loco *transcript which is expressed during development.

### G-protein α subunits are also expressed in the adult reproductive system

LOCO is a regulator of G-protein signalling and has been shown to interact with various Gα subunits [14,17]. Searching the *Drosophila *genome and various EST databases we found several Gα transcripts and proteins. We wanted to investigate if different Gα subunits were expressed in the testis providing potential binding partners for the isoforms of LOCO that are generated. The three Gα genes we choose to analyse were *G-oα47A, Gα73B*, and *Gα49B*.

*G-oα47A *the *Drosophila *homologue of the mammalian Goα, is needed for embryonic development [18-20] and has more recently been shown to contribute to asymmetric cell division [21]. Furthermore it is expressed in the nurse cells and oocyte and is present in various adult nerve cells [22]. *Gα73B *encodes a further Gα subunit called Gfα, it is expressed in the embryonic midgut and in the nurse cells after which it is transported to the oocyte [23]. Gα49B, a Gq subunit involved in phospholipase C activation, is involved in the *Drosophila *visual system [24,25]. Gα49B is known to be expressed in the adult testis [26], and thus acted as a positive control. PCR showed that *G-oα47A, Gα73B *and Gα49B were expressed in the testis (Figure 2D). This raises the possibility that in the adult testis there are additional Gα subunits that may interact with LOCO.

### Analysis of mutant phenotypes

**Figure 3:**
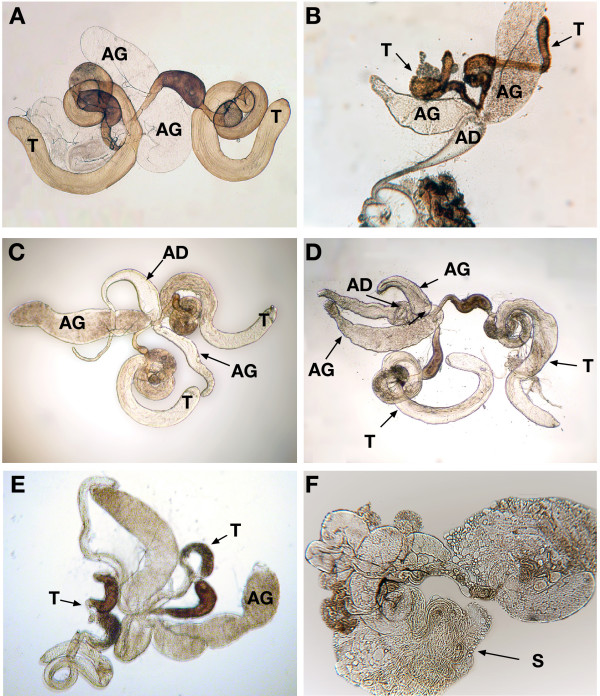
**The adult reproductive tissue from the *loco *mutants**. **A**: Adult reproductive system of *OrR Drosophila melanogaster*. **B**: The testes of the mutant *loco*^*318*^. The testes in this fly seem much smaller and thinner than *OrR*. **C**: The male reproductive tissue of *loco*^*358*^. The accessory glands are small and differ in size; furthermore, the anterior ejaculatory duct appeared swollen. **D**: The male reproductive tissue of the *loco*^*358 *^mutant heterozygous with *OrR*. Both testes have formed and the accessory glands seem fuller than the homozygous mutant. The anterior duct seems less swollen and the posterior end looks fuller. The morphology is still very unlike wild-type testes however these flies are fertile. **E**: The male reproductive tissue of *loco*^*387*^. The testes remain unwound and are much smaller than the accessory glands. The accessory glands are not as full as *OrR*. **F**: The male reproductive tissue of *loco*^*387 *^is flattened and ruptured and spermatid bundles are observed. T: Testis, AG: accessory gland, AD: anterior ejaculatory duct and S: sperm.

We have shown that *loco *and various Gα subunits are expressed in the testis and have identified, genetically, that mutation in *loco *can lead to significantly reduced fertility in males. We therefore went on and analysed adult gonad morphology in the *loco *mutants. All of the *loco *mutants had abnormal reproductive systems and showed a variety of defects (Figure 3B–E). The phenotype was almost 100% penetrant, there was a range in severity within the same line, and only the very occasional male reproductive system was normal. Often the accessory glands were abnormal and the ejaculatory ducts appeared swollen. Neither testes from virgin wild-type males or aged wild-type males show this peculiar phenotype, suggesting that this phenotype was not attributable to the mating status (data not shown). There were no obvious differences between the mutant lines, which may be attributable to all mutants being in the same complementation group. The testes, even those very abnormal in shape and size, contained mobile sperm (Figure 3F). This suggested that the differentiation of the sperm does not require the *loco *isoform affected in this group of mutants, but possibly differentiation and morphogenesis of the derivatives of the genital discs and gonad are altered and often fails in these mutants. Identification of motile sperm in the male mutants may explain why some offspring were produced; it seems likely that the abnormal morphology of the gonads is the causative effect of reduced fertility.

### Expression of *loco *during male development

**Figure 4:**
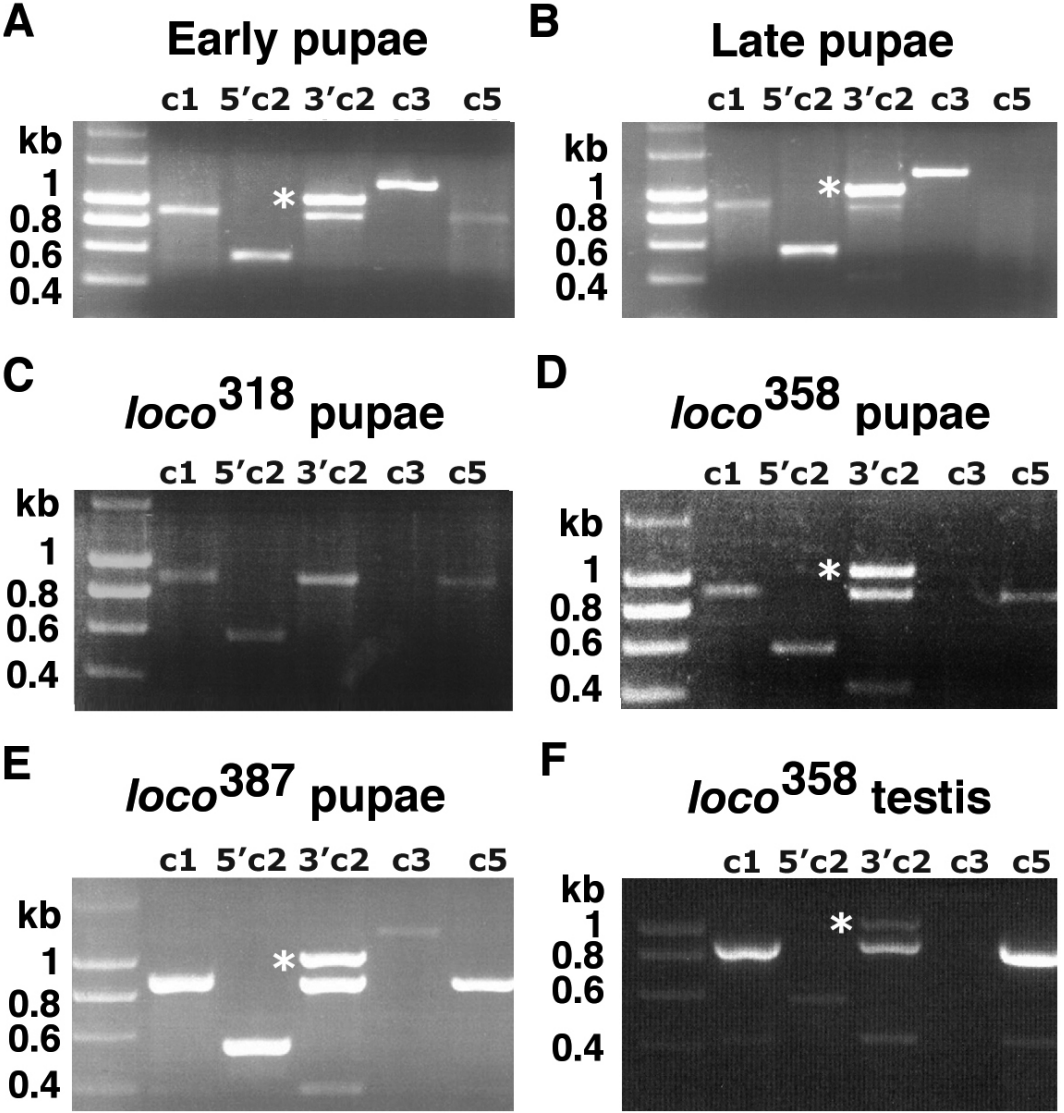
**Identification of the various *loco *transcripts in pupae and testes**. **A**: Transcripts *loco c1*, *loco c2*, *loco c3*, and *loco c5 *were detected in early pupae. **B**: The transcripts *loco c1*, *loco c2*, and *loco c3 *were detected in late staged pupae, however *loco c5 *was not. **C**: Expression of *loco c1 *was detected in *loco*^*318 *^as was the 5' end of *loco c2*. However the 3'end of *loco c2 *(3' c2) was not detected in *loco*^*318 *^as only the non-specific band was produced (Sequencing and alignment showed this band to be CaBP1). Expression of *loco c3 *was also absent in *loco*^*318*^, however expression of *loco c5 *remained unaffected. **D**: Only expression of *loco c3 *was lost in the pupae of the mutant line *loco*^*358*^. **E**: All *loco *transcripts were detected in the pupae of the mutant line *loco*^*387*^. **F**: Expression of *loco c3 *was lost in the testes of the mutant line *loco*^*358*^. The true PCR product of *loco c2 *is marked with an asterisk.

The phenotypes of the mutant testes and reproductive system suggested that organogenesis and not necessarily spermatogenesis was abnormal. Since differentiation of the testis and reproductive system occurs during metamorphosis we investigated the expression of *loco *in early and late pupae (Figure 4A and 4B). We divided the pupae into early pupae and late pupae and performed RT-PCR on the various *loco *transcripts (Figure 1A). The *loco c1*, *loco c2*, and *loco c3 *transcripts were expressed during the early stages and late stages of pupal development. The newly identified transcript, *loco c5*, was found to be differentially expressed and seemed to be detectable only in early staged pupae (Figure 4A and 4B).

We have now shown that *loco *is expressed during metamorphosis. In order to ascertain whether *loco *is expressed during organogenesis of the male gonad, the original P element insertion line, which contains *GAL4 *in the *loco *gene [15], was crossed to a *UAS-lac*Z reporter strain. β-galactosidase activity was detected in the larval male gonads (Figure 2B, arrow) as well as in the fat body tissue surrounding the gonad. The observed β-galactosidase activity in the gonad confirms that expression of the *loco *gene takes place in the male gonad during development.

### Expression of *loco *in mutant pupae and testes

We have demonstrated that *loco *is expressed in the developing male and adult gonads, furthermore we have shown that loss of *loco *expression resulted in reduced fertility and testis with abnormal morphology. In order to ascertain which transcript was affected by the mutation we performed RT-PCR for *loco c1*, *loco c2*, *loco c3 *and *loco c5 *in the mutant pupae of three of the male semi-sterile lines, *loco*^*318*^, *loco*^*358*^, and *loco*^*387 *^(Figure 4C–E). We detected *loco c1*, the 5'end of *loco c2 *and the *loco c5 *transcripts in the *loco*^*318 *^mutant. However *loco*^*318 *^completely lacked expression of the *loco c3 *transcript (Figure 4C). Furthermore *loco*^*318 *^only expressed the 5'end of *loco c2*. The 3'end of *loco c2 *was not detected (Figure 4C, 3'c2) as only the 0.9 kb non-specific band was detected. Pupal expression from the *loco *gene in the *loco*^*358 *^mutant produced *loco c1*, *loco c2 *and *loco c5*, however like *loco*^*318 *^the *loco*^*358 *^mutant lacked *loco c3 *(Figure 4D). Pupal expression of *loco c1*, *loco c2*, *loco c3 *and *loco c5 *was detected in the *loco*^*387 *^mutant (Figure 4E). The testis expression of *loco*^*358 *^was also found to lack any detectable *loco c3 *transcript.

The RT-PCR analysis was carried out on multiple different RNA samples and the same result was consistently achieved. Whenever the quality of the RNA was checked on a formaldehyde agarose gel it was found to be intact (data not shown). Furthermore detection of *loco c1*, *loco c2 *and *loco c5 *from the same cDNA samples which lacked *loco c3 *suggests that the RNA transcribed from the *loco *gene was intact. Taken together this data suggests that there is a requirement for *loco c3 *in adult reproductive tissue.

### Analysis of the genomic sequence in the mutant lines

To gain further insight into molecular nature of the *loco *mutations, PCR was performed on genomic DNA isolated from the original c139 strain [15]. PCR utilising a P element primer directed to the 5' end of the P element (pgawb5a inv) in combination with a gene specific primer revealed that the P element was in reverse orientation to the *loco *gene (the 5' end of the P element was orientated toward the 3'end of the *loco *gene). Sequencing of the PCR product revealed that the P element had inserted 322 bp upstream of exon I-1 (Figure 1A).

PCR analysis of the *loco*^*318 *^mutant revealed that the P element primer directed to the 5' end of the P element (pgawb5a inv) worked in combination with gene specific primers in the forward and reverse orientation. This suggested that there was more than one P element present in *loco*^*318 *^and that they were in the opposite orientation to one another. Direct sequencing of these products revealed that one P element was present at the original position (322 bp upstream of exon I-1) and the second P element was present at the same position but in the opposite direction (Figure 1A). The sequencing of the PCR products from *loco*^*318 *^also revealed a 9 bp duplication of genomic DNA on either side of the P elements (Figure 1A). It is possible that two P elements 322 bp upstream of exon 2 alters pre-mRNA length such that there is premature dissociation of RNA polymerase II (RNAPol II), hindrance of the folding of the pre-mRNA molecule which prevents the joining of the splice sites, or it may disrupt important splice factor binding sites.

The P element primer used to reveal the position of the P element in the mutant line *loco*^*318 *^failed to produce PCR products in the mutant line *loco*^*358*^. PCR across the original insertion site revealed that the P element had excised and had not deleted any genomic sequence (Figure 1B). The mutant line *loco*^*358 *^expresses *white*, it lacks expression of *loco c3 *(Figure 4D) and the mutation genetically maps to the *loco *gene (Table 2). This suggested that a partial P element, which lacks the P element primer site, was present in the *loco *gene. PCR with the P element primer and FP8 produced a fragment of 2 kb, suggesting that the P element was 2 kb upstream of exon 2. Genomic PCR was performed across all introns in the *loco *gene. PCR failed only between exon I-1 and exon 2, whereas a PCR product across this region in wild-type genomic DNA was detected (data not shown). This further suggested that the partial P element in the mutant line *loco*^*358 *^was located between exon I-1 and exon 2 and thus produced a PCR product too long to be detected by the PCR programme.

Direct sequencing of the insertion site in *loco*^*387 *^revealed that no sequence had been deleted (Figure 1B). The *loco*^*387 *^line was found to express *loco c1*, *loco c2*, *loco c3*, and *loco c5 *(Figure 4E). The reduction in male sterility when *loco*^*387 *^is hemizygous with *Df15*, *Df17 *or *loco*^*Δ13 *^was statistically significant (Table 2) and the adult reproductive tissue of *loco*^*387 *^is morphologically abnormal (Figure 3E). This suggests that a mutation resides within the *loco *gene. Large rearrangements can occur during P element mobilisation. The PCR product across the *loco *insertion site was approximately 500 bp, therefore if a large inversion or rearrangement had occurred in *loco*^*387 *^it would not be detected by this simple PCR. These data alongside the genetic data strongly suggests that the mutations lie within the *loco *gene.

## Discussion

We isolated a number of homozygous lethal mutant lines of *loco*. These lines die at a variety of developmental stages, however, among them four lines were able to generate some homozygous adult males, which were semi-sterile. We suggest that this is most likely to be due to a failure of the correct morphogenesis of the testis and reproductive organ derivatives of the larval gonad. This adds another role to the wide range of developmental decisions that are known to be dependent upon *loco*. Granderath *et al*., (1999) [14] showed that *loco *mutants died as embryos showing abnormalities in the contacts between glial cells [14]. Our previous studies illustrated that there is a requirement for *loco *in cytoplasmic dumping from the nurse cells to the oocyte and that *loco *is required for correct patterning of the eggshell and embryo [15]. There is also a large maternal supply of *loco *in the embryo probably explaining why the embryos die so late in embryogenesis. Finally, it was shown more recently that *loco *contributes to asymmetric cell division of neuroblasts [13]. These findings suggest that G-protein signalling may be important at wide variety of developmental stages in *Drosophila*.

The *loco *gene expresses several splice variants *loco c1, loco c2, loco c3, and loco c4 *[13-15]. Here we describe the expression of a fifth transcript, *loco c5*. We have analysed the expression of *loco c1*, *loco c2*, *loco c3*, and *loco c5 *in the wild-type testis and developing pupae and show that there is developmental regulation of *loco c5 *expression during morphogenesis. In addition we show that several G proteins are expressed in the male gonads and are therefore potential binding partners for the various LOCO isoforms. It is possible that the protein isoforms, expressing different conserved domains, will have different binding specificities and preferences for different G-proteins [27-31]. The G protein Gαi (G-oα65A) binds to *loco c2 *[14] and it is also co-expressed with *loco *in a variety of cell types [16,32]. We have shown by PCR that other Gα subunits are expressed (Goα47A, Gα49B, and Gα73B) in the testis and thus there is potential for LOCO to interact with other Gα subunits.

The analysis of the final morphology of the adult reproductive system in all of the flies analysed, strongly suggests that there is a failure in male gonad and genital morphogenesis It is possible that *loco c3 *expression could be the underlying reason for this phenotype. However the variability in testes morphology between flies may hint that there is some level of redundancy between the *loco *transcripts. Thus, whilst *loco *is clearly essential, a lack of or reduction of *loco c3 *expression does not cause a complete failure of gonad and genital differentiation. The *loco *mutants we isolated still express several *loco *transcripts, so further mutants will be needed which disrupt different transcripts or sets of transcripts to discover the role of *loco *and G-protein signalling in spermatogenesis and to further investigate it in imaginal discs and in the somatic cells of the gonad.

## Conclusion

We show that all of the known *loco *spliceforms are expressed in the pupae and testis. In addition to this we have identified a fifth loco transcript, *loco c5*. We also show that there are a variety of Gα proteins expressed in the testis that may interact with the various LOCO isoforms. We have generated a set of new alleles of *loco *that affect the expression of specific *loco *transcripts. These deletions seem to be highly deleterious to *Drosophila*, as only a few adults hatch and the majority die as larvae. Mutant pupae and adult gonads of the few males that hatch show a loss of *loco c3*. We propose that *loco c3 *is needed for correct morphogenesis of the male gonad and the reproductive system derived from the male genital disc during metamorphosis. The role we have observed for *loco *in morphogenesis is in some ways similar to its role in glial cells where it has been proposed that G-protein signalling is important for shape changes [14]. Although the reproductive system is derived from the genital disc and the testis from the gonad, both tissues are affected. It, therefore, seems likely that *loco *is involved in cell-cell interactions during evagination and morphogenesis. During these processes cell and tissue shape changes are crucial.

These results support the well-documented findings that G-protein signalling is crucial throughout development. An extensive investigation is needed to identify the binding specificities of different *loco *isoforms, the temporal and spatial distribution of different *loco *transcripts and which Gα subunits co-localise with *loco *in the gonad and genital discs and in the adult male testis. With this information it will be possible to design genetic and molecular experiments to investigate the developmental mechanisms in which *loco *participates.

## Methods

### Stocks

Wild-type flies were *OrR*. *Df(3R)17D1 Df(3R)15CE1*, *loco*^*Δ13 *^*and loco *^*T1 *^were obtained from Christian Klambt. The original P insertion line was c139; it has an insertion of *GAL4 *in the *loco *gene [15]. The mutant lines were generated and described in Pathirana *et al 2*001 [15]. All *Drosophila *strains were raised on standard cornmeal-yeast-agar medium at 25°C.

### β-galactosidase staining of testes and gonads

Testes and male gonads were dissected from the progeny from c139 crossed to the UAS-*lac*Z reporter line. Staining was carried out as described by Deng *et a*l 1999 [33].

### DNA sequencing

The dideoxy chain determination method was used initially in the form of a Sequenase 3.1 kit (US Biochemicals), followed by automated sequencing on Perkin Elmer ABI 373A and 377A machines using dye labeled primers, then dye labeled terminator reactions. Sequenced fragments were assembled using GCG and GENE-JOCKEY software. Sequence analysis was done with GCG GAP, MAP, FASTA, TFASTA and PILEUP software. Conserved domains were predicted using SMART [34] and the NCBI conserved domain database [35].

### Reverse transcription (RT)-PCR

Reverse transcription-PCR was carried out as described in Deng *et a*l 1999 [33]. The primers used to identify transcripts were:

LOCOFPB1 GTGGATCCAGCTCCAATAGCCGCAATCTC

LOCORPE4 CGGAATTCACACTTTCTCCTCCTGCAC

LOCOFPB5 ATGGATCCGCAGTTCATACGATCACAACGC

LOCOFPB6 ATGGATCCTGCCATTGTATTGCCCAC

LOCOFPB8 GTGGATCCTTGTCTCAGCAGCTCGTCC

LOCOFP9 ATGGATCCACGAATCTCACATCCACCG

LOCOFP10 TATTCCCATCGCATTGCCC

The primer pairs used to identify Gα subunits were:

Gα 73B (G-αF), accession number: CG12232-RA

forward ATTGGTGGGTCAGATGGGAG

reverse TCCTCAGGCCCCCACCAACTGTGATCTAG

Gα 49B (Gqα-3) Accession number: U31092

forward CGGGAAGTCCACATTCATC

reverse CGTGTGCTACAGATACGGA

Goα 47A (G-αO) accession number: M30152

forward GTCCCCTGACGATTTGCTTCC

reverse CAAACACAAGCGCCAACATT

### Preparation of and analysis of genomic DNA

Homozygous mutants were homogenized in 100 mM Tris pH 8.5, 80 mM NaCl, 50 mM EDTA pH 8, 0.5% (w/v) sucrose, 0.5% (w/v) SDS. The samples were incubated at -70°C for 30 minutes and then 65°C for 30 minutes. Potassium acetate was added to a final concentration of 1 M, the cell debris was discarded and the supernatant precipitated with isopropanol.

Primers designed to the intronic sequence were:

LOCOGFP1 AGTAAGCAGCGCACATGCAC

LOCOGRP2 GGCAGAAAGCGAAAACGTGAC

LOCOGFP3 CCAGAATACCCATCGCAAG

LOCOGRP4 TCAGACGGGCACGATAAAC

The P element primer used was designed to the inverted repeat:

pgawb5a inv CCACCTTATGTTATTTCATCATG

## Authors' contributions

LM conducted molecular analysis of transcripts and gene organisation and helped with manuscript preparation and analysis of results. SP designed and carried out the screen and helped with the supervision of two students analysed the mutants; MB helped with the design and direction of the project, the preparation of the manuscript and analysis of results. TT helped with molecular analysis of mutants. PC helped with analysis of mutants by complementation testing. FS isolated of some of the mutants used. WS helped with isolation of some of the mutants used with FS. KR helped with the maintenance of flies, repeats of crosses and dissections. All authors read and approved the final manuscript.
